# Spatial Patterning Analysis of Cellular Ensembles (SPACE) enables statistically robust discovery of complex spatial organization at the cell and tissue level

**DOI:** 10.1101/2023.12.08.570837

**Published:** 2023-12-10

**Authors:** Edward C. Schrom, Erin F. McCaffrey, Andrea J. Radtke, Emily Speranza, Leanne Arakkal, Nishant Thakur, Spencer Grant, Ronald N. Germain

**Affiliations:** 1 Lymphocyte Biology Section, Laboratory of Immune System Biology, National Institute of Allergy and Infectious Diseases, National Institutes of Health, Bethesda, MD 20892-1892, USA.; 2 T-Lymphocyte Biology Section, Laboratory of Parasitic Diseases, National Institute of Allergy and Infectious Diseases, National Institutes of Health, Bethesda, MD 20892-1892, USA.; 3 Center for Advanced Tissue Imaging, Laboratory of Immune System Biology, National Institute of Allergy and Infectious Diseases, National Institutes of Health, Bethesda, MD 20892-1892, USA.; 4 Florida Research and Innovation Center, Cleveland Clinic Lerner Research Institute, Port Saint Lucie, FL 34987, USA.; 5 Center for Alzheimer’s and Related Dementias, National Institute on Aging, National Institutes of Health, Bethesda, MD 20892-1892, USA.

## Abstract

Spatial patterns of cells and other entities drive both physiologic and pathologic processes within tissues. While many imaging and transcriptomic methods document tissue organization, discerning these patterns is challenging, especially when they involve multiple entities in complex arrangements. To address this challenge, we present Spatial Patterning Analysis of Cellular Ensembles (SPACE), an R package for analysis of high-plex tissue images generated using any collection modality. Unlike existing platforms, SPACE detects context-dependent associations, quantitative gradients and orientations, and other organizational complexities. Via a robust information theoretic framework, SPACE explores all possible ensembles – single entities, pairs, triplets, and so on – and ranks the strongest patterns of tissue organization. Using lymph node images for which ground truth has been defined, we validate SPACE and demonstrate its advantages. We then use SPACE to reanalyze a public dataset of human tuberculosis granulomas, verifying known patterns and discovering new patterns with possible insights into disease progression.

## INTRODUCTION

Biological tissue function emerges from the local interactions of diverse cells and structures. Whether these interactions occur via direct contact or paracrine signaling, they are determined by the spatial arrangement of the involved entities. Thus discovering new spatial patterns can reveal new mechanisms of tissue (dys)function [Baccin et al 2020, Saviano et al 2020, Lewis et al 2021, Rendeiro et al 2021, Germain et al 2022].

Many methods exist to capture the spatial arrangements of deeply phenotyped cells and structures. Protein-targeted imaging methods, using fluorescence [Lin et al 2016, Radtke et al 2022], sequencing [Black et al 2021], or heavy metal isotopes [Giesen et al 2014, Keren et al 2019], capture 30–100 molecular parameters at submicron resolution. Transcript-targeted imaging methods capture hundreds of molecular parameters at similar resolution [Eng et al 2019, Xia et al 2019, Moffit et al 2022], or the entire transcriptome at lower spatial resolution [Stahl et al 2016, Rao et al 2021]. Multimodal techniques combine the advantages of several methods [Vandereyken et al 2023]. In each case, dozens to hundreds of cell types and structures can be identified, and the spatial patterns created by these many elements are too numerous for manual exploration.

To analyze high-plex spatial data, many computational platforms have been introduced [Schapiro et al 2017, Goltsev et al 2018, Stoltzfus et al 2020, Dries et al 2021, Bhate et al 2022, Bartolomeazzi et al 2022, Warchol et al 2023]. Existing platforms essentially perform two classes of analyses. The first examines pairwise associations based on the frequency of spatial co-occurrence [Schapiro et al 2017], spatial correlation [Stoltzfus et al 2020, Warchol et al 2023], or nearest distances between individual cells [Bartolomeazzi et al 2022]. These analyses do not directly address patterns involving three or more cell types. Moreover, they only detect linear associations, i.e., associations that occur consistently throughout a tissue sample, but not nonlinear associations, i.e., context-specific associations that differ across subregions of a tissue sample. The second class of analysis uses machine learning (e.g., K-means) or topic modeling to define tissue microenvironments (MEs) of consistent cell type compositions [Goltsev et al 2018, Chen et al 2020, Schurch et al 2020, McCaffrey et al 2022, Nirmal et al 2022, Radtke et al 2023]. While this approach uses all cell types instead of just two, it often lacks statistical validation: the requested number of MEs will be found, whether or not their compositions are optimally distinguished from random assortment [Halkidi et al 2001, Baya & Granitto 2013]. Moreover, classification of discrete MEs ignores continuous gradients of cellular abundance. Finally, both classes of analyses rely on tabulated centroid locations of segmented cells, requiring customized approaches for irregularly shaped cells, acellular structures, or raw measurements of molecular expression.

**Figure 1.**
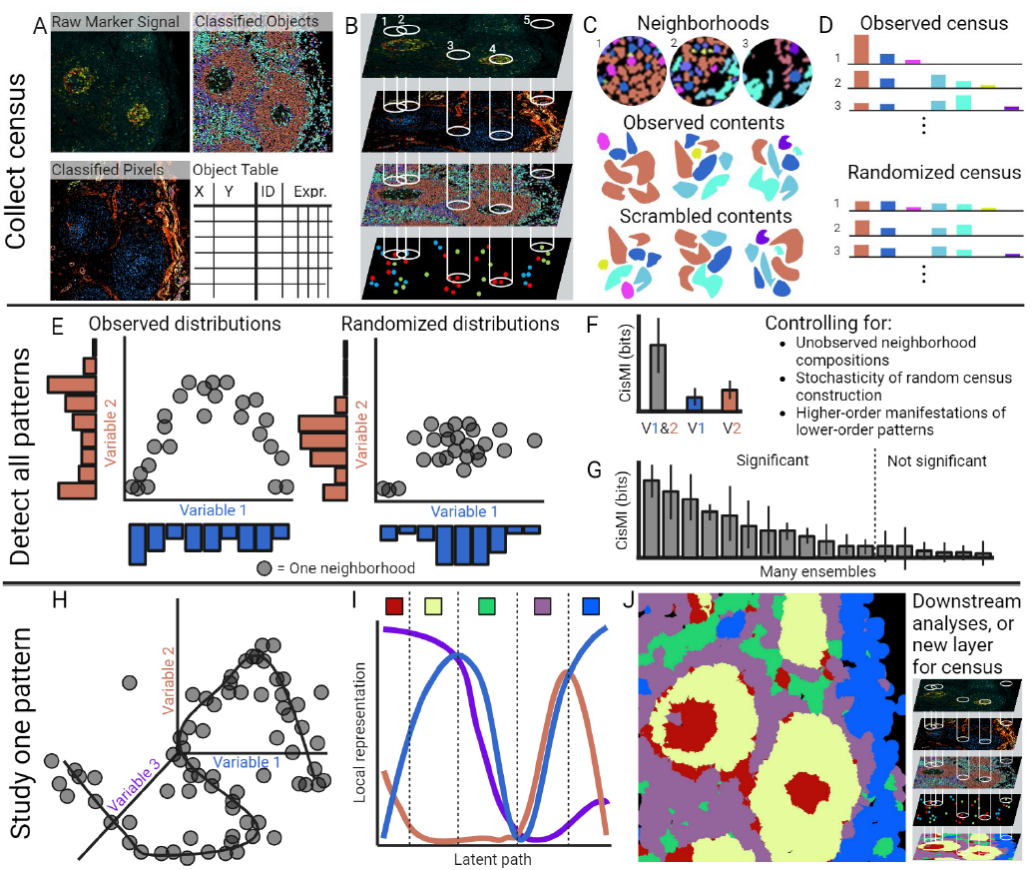
SPACE workflow. A) SPACE accepts images of molecular expression, images of segmented and classified cells, images of classified pixels, and centroid tables of cellular phenotypes and/or mean molecular expression. B) Neighborhoods are drawn through each layer of provided data. C) Neighborhood contents are tabulated, as are contiguous patches of each variable, which are scrambled to form randomized neighborhoods. D) True neighborhood contents form the observed census, while scrambled neighborhood contents form randomized censuses. E) For a given ensemble, observed and randomized distributions of co-occurrence are generated. F) The KL divergence between the observed vs. randomized version of each distribution is calculated. G) KL divergences are adjusted for several factors to yield cisMI, which is then used to rank the ensembles with the strongest spatial patterning. H) For a given ensemble, a self-organizing map learns the shape of non-random patterning as a curve in high dimension. I) The high dimensional curve is projected onto the covariation plot revealing specific features of the ensemble’s patterning. J) Manually-defined regions are mapped back onto the tissue for further rounds of SPACE analysis. Created with Biorender.com.

To overcome these limitations of existing approaches, we present SPACE (Table 1). SPACE is a novel information-theoretic algorithm that systematically detects, statistically validates, and thoroughly characterizes spatial patterns of arbitrary complexity in imaged tissue samples. SPACE operates on raw molecular expression data, classified pixels, spatial maps of cellular segmentation, and/or centroid data simultaneously (Fig 1a–d), broadening the scope of entities that can be analyzed. For any combination of these entities, SPACE leverages information theory to quantify the deviation from random patterning in a metric we call “cis mutual information” (cisMI) (Fig 1e–g, Fig S1). Simple assumptions of parsimony allow SPACE to perform an exhaustive exploration and yet discern the top spatial patterns most likely to reveal interesting biology. Specific patterns are then characterized in detail, revealing not only co-assortment but also gradients, orientations, and other complex features that may be context-specific (Fig. 1h–j). SPACE is available as an open-source R package.

We demonstrate and validate SPACE on data from different imaging modalities, host species and tissues, and input data formats. We focus on the mouse lymph node, where ground-truth spatial patterning is known, as well as a recent study of human tuberculosis (TB) granulomas [McCaffrey et al 2022]. Although TB causes 1.6 million human deaths annually [WHO Global Tuberculosis Report 2022] and is widely studied, mechanisms of disease progression are not fully understood [Flynn & Chan 2022]. In addition to rediscovering known patterns in these data, SPACE provides a new level of exploratory power that translates the enormous complexity of high-plex spatial data into a small set of candidate spatial patterns most likely to reveal important underlying biological processes.

## RESULTS

### SPACE Detects Known Patterns and Gradients in the Mouse Lymph Node

**Figure 2.**
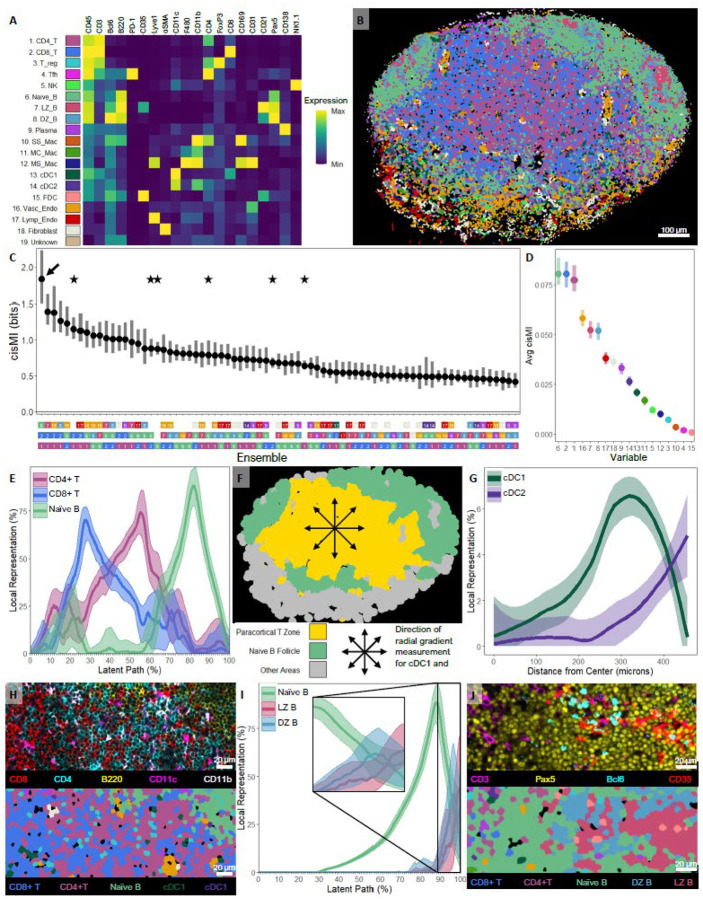
Validation of SPACE on a mouse LN. A) Marker expression profiles of 19 segmented cell types. B) Segmentation image used in SPACE analysis. C) SPACE quantification of cisMI for the 75 most significantly non-randomly patterned ensembles. D) Average cisMI for each cell type across all significant ensembles. E) Covariation plot for CD4+ T cells, CD8+ T cells, and naïve B cells. F) Zone image obtained from partitioning the covariation plot into T- vs. B-cell dominated areas. G) Gradients of cDC1 and cDC2 abundance from the interior to the exterior of the mapped T zone. H) Visual example of the CD4+ T vs. CD8+ T and cDC2 vs. cDC1 gradients in the T cell zone, with respect to the B follicle. I) Covariation plot for naïve B, LZ B, and DZ B cells. J) Visual example of the orientation of GCs with the DZ facing the interior of the naïve B follicle and the LZ facing the exterior.

To compare SPACE output against ‘ground-truth’ for a well-studied tissue, we used a 42-plex image of a mouse popliteal lymph node (LN) captured with the IBEX protocol [Radtke et al 2020] [Fig. S2a]. Using CellPose 2.0 [Pachitariu and Stringer 2022], we segmented individual cells [Fig. S2b]. To restrict our focus to well-studied cellular subsets, we chose 20 lineage markers to define canonical expression profiles [Table S1] and deployed a semi-supervised pipeline [Fig. S2c–h] to classify the cells into 19 types [Fig. 2a] and map them onto a segmentation mask [Fig. 2b]. We censused 3000 neighborhoods of radius 20 μm on this mask and measured cisMI for every ensemble of one, two, or three cell types. Of the possible 1159 ensembles, 498 (43.0%) have significant patterning, and these are ranked by cisMI (Fig. 2c). Individual cell types are also ranked by average cisMI (Fig 2d). Performing the same analysis on a table of cell centroids yields similar, albeit less powerful, results (Text S1, Fig S3).

The three most strongly patterned cell types on average are CD4+ T cells, CD8+ T cells, and naïve B cells (Fig. 2d). These populations define the largest microanatomical zones of the LN – the paracortical T zone and primary B follicles. These zones can be explained purely by self-clustering of each cell type, co-clustering of CD4+ and CD8+ T cells, and separation of naïve B cells from T cells (Fig. 2c, stars). Even controlling for these patterns, the strongest ensemble includes all three populations (Fig. 2c, arrow), indicating that their patterning encodes more than just the obvious microanatomical zonation.

We investigated this tripartite pattern using a covariation plot (Fig. 2e). In accordance with the general separation of the T zone and B follicles, the covariation plot shows separate regions where T cells vs. naïve B cells dominate (Fig. 2e, 15–65% along the x-axis vs. 65–100%). Additionally, within the T zone, CD4+ and CD8+ T cells form opposing gradients, where CD4+ T cells dominate closer to the main B follicle, whereas CD8+ T cells dominate farther away. Although the visual manifestation of this pattern depends on the slicing angle of the LN (Fig S4), it remains that CD4+ and CD8+ T cells co-occur at large but oppose one another specifically within the B-cell-devoid T zone. This nonlinear context-dependent pattern, which requires all three cellular partners to detect, was discovered recently [Baptista et al 2019] using manual histo-cytometry analyses [Gerner et al 2012], and SPACE recovers via automated exploration. Additionally, after mapping the B follicle and T zone (Fig. 2f), SPACE finds that Type 1 dendritic cells (cDC1s) peak in abundance closer to the center of the T zone, whereas cDC2s peak in abundance at the edge (Fig 2g), as originally discovered [Gerner et al 2012, Baptista et al 2019]. The combined organization of lymphocytes and DCs discovered by SPACE is clear from raw marker expression (Fig. 2h).

Finally, we chose a weaker ensemble: naïve B cells, light zone (LZ) germinal center (GC) B cells, and dark zone (DZ) GC B cells (Fig 2c, square). The covariation plot shows that these cell types are concentrated in three nearby zones, as expected. Additionally, DZ B cells are proximal to the bulk of the naïve B follicles which borders the T zone, whereas LZ B cells are distal (Fig. 2i). This is visually apparent (Fig. 2j). While the separation of the naïve B follicles, GC LZ, and GC DZ can be explained in terms of single or paired cell, positive cisMI for the tripartite pattern is statistical evidence that GC orientation within the B follicles, which requires all three cell types, is also significantly non-random. Neither GC orientation nor the T cell or cDC gradients are readily detectable with existing spatial analysis approaches (Text S2, Fig S5–S6).

### SPACE Detects Known and New Patterns in Human TB

**Figure 3.**
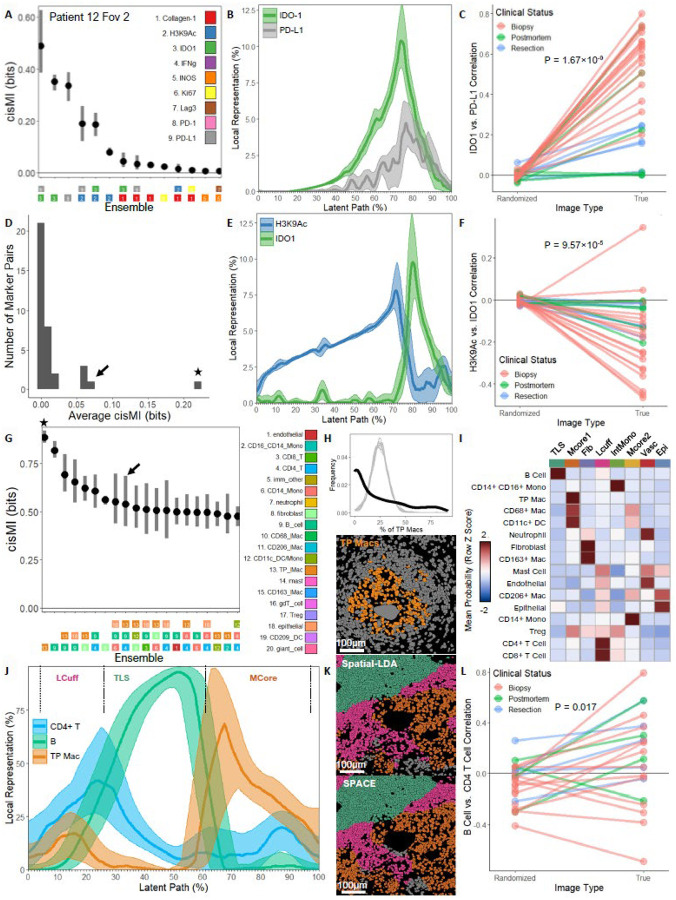
SPACE re-analysis of human TB granulomas. A) SPACE quantification of cisMI for all significant ensembles of functional markers in Patient 12 FoV 2. B) Covariation plot for PD-L1 and IDO1 in Patient 12 FoV 2. C) Correlation of PD-L1 and IDO1 expression in the true vs. randomized images across the full data set (paired T test). D) Average cisMI across all FoVs for each pair of functional markers, including PD-L1 and IDO1 (highest average cisMI) as well as H3K9Ac and IDO1 (second-highest average cisMI). E) Covariation plot of H3K9Ac and IDO1 in Patient 12 FoV 1. F) Correlation of H3K9Ac and IDO1 in the true vs. randomized images across the full data set (paired T test). G) The 25 strongest patterns among segmented cell types in terms of cisMI averaged across the full data set. H) Observed (black) vs. randomized (gray) distribution of TP macrophage abundance in 50μm neighborhoods in Patient 10 FoV 1. I) Compositions of the MEs discovered by Spatial-LDA in the original study, defined by the prevalence of 16 different segmented cell types. J) Covariation plot for TP Macs, B cells, and CD4+ T cells in Patient 8 FoV1, with the gates used to define the Lcuff, TLS, and Mcore1 MEs. K) Lcuff, TLS, and Mcore1 MEs for Patient 8 FoV 1 as drawn by Spatial-LDA vs. SPACE. L) Correlation of B cell and CD4+ T cell abundance specifically in 50μm neighborhoods where TP Macs occur outside the Mcore1 ME in the true vs. randomized images across the full data set (paired T test).

We next reanalyzed a publicly available data set of human TB granulomas. Using a 37-plex MIBI-TOF panel, two 500μm × 500μm fields of view (FoVs) were imaged from each of 15 human TB patients [McCaffrey et al 2022]. Of these patients, nine were undergoing diagnostic biopsies, three were undergoing therapeutic resections, and three were sampled postmortem. The original study uncovered consistent patterns of granuloma organization across clinical status. One of the strongest is the co-expression of immune-inhibiting molecules PD-L1 and IDO1 at the cellular level. After censusing 5 μm neighborhoods to capture single cells, we used SPACE to all pairs of the nine functional markers included in the original study. In many individual FoVs, SPACE finds that PD-L1 and IDO1 are the most strongly patterned pair of markers (Fig. 3a), due to co-expression (Fig. 3b). This is consistently observed across the full data set (Fig. 3c). Although PD-L1 and IDO1 consistently form the strongest spatial pattern (Fig. 3d, star), SPACE finds other marker pairs with average cisMI > 0 across the 30 FoVs (Fig. 3d, arrow), which were undetected in the original study. For example, H3K9Ac and IDO1 are negatively correlated in their expression, both in representative FoVs (Fig. 3e) and consistently across the data set (Fig. 3f). Because negative pairwise correlations are typically detectable by other approaches, this likely reflects SPACE’s advantage of analyzing images of raw signal rather than tables of average cellular expression.

We also used SPACE to examine spatial patterns among the 20 segmented cell types defined in the original study. After censusing 50 μm neighborhoods to match the original study, we quantified cisMI for every ensemble of one, two, or three cell types in each FoV and then averaged across FoVs to find the strongest cellular patterns in the full data set (Fig. 3g). Several of the strongest patterns are formed by single cell types, e.g. CD11b/c+ CD206+ macrophages (“TP Macs”), which self-cluster more than expected by random chance (Fig. 3h, star). This agrees with the Spatial-LDA clustering analysis of original study (Fig. 3i), which found that TP Macs were largely restricted to a single ME, termed “Myeloid Core 1” or “Mcore1.”

We also studied the strongest tripartite pattern for which all three cell types were present in ≥50% of images, which includes TP Macs, B cells, and CD4+ T cells (Fig. 3g, arrow). In an illustrative FoV, these populations dominate in three separate areas of the granuloma (Fig. 3j). This agrees with the original Spatial-LDA analysis, which found that these three cell types were each restricted to a different ME (Fig. 3i). Gating on the covariation plot reveals MEs nearly identical to those discovered by Spatial-LDA (Fig. 3k). However, mere separation into distinct MEs only requires single and pairwise patterns: each cell type self-clusters, and each pair of cell types is mutually exclusive. The significant cisMI of the tripartite pattern indicates that other features of their spatial patterning are also important. Although B cells and CD4+ T cells dominate in separate MEs, they appear positively correlated specifically in non-Mcore1 niches where TP Macs are present in small numbers (Fig 3j, 10–20% on the x-axis). This context-dependent co-occurrence of B and CD4+ T cells is consistent across the full data set (Fig. 3l). This suggests that in TB granulomas, TP Macs at the edges of or outside Mcore1 can unite B and CD4+ T cells that would otherwise separate from one another. Thus, SPACE recovers patterns found by Spatial-LDA while also discovering context-specific gradients that are missed by clustering algorithms. Even patterns from the original study that integrate raw marker expression and cellular phenotypes/MEs are recovered and refined by SPACE (Text S3, Fig. S7).

### SPACE Discovers Cellular Patterns Associated with TB Disease Severity

**Figure 4.**
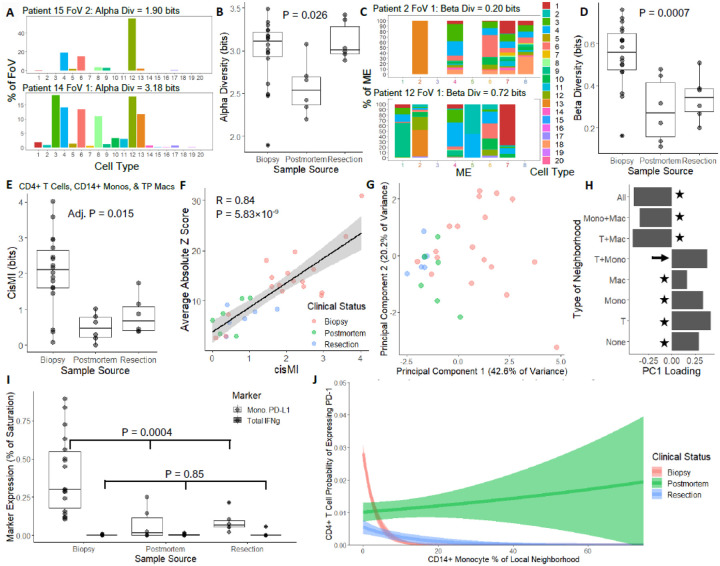
Novel correlates of TB disease progression discovered with SPACE. A) Example FoVs with low and high cellular alpha diversity. B) Comparison of cellular alpha diversity across clinical statuses (ANOVA). C) Example FoVs with low and high cellular beta diversity across MEs. D) Comparison of cellular beta diversity of MEs across clinical statuses (ANOVA). E) CisMI for the tripartite ensemble of CD4+ T cells, CD14+ monocytes, and TP macrophages compared across clinical statuses (ANOVA). F) Correlation of average absolute Z scores for the prevalence of each neighborhood category with cisMI. G) PCA of FoVs using the Z scores for each neighborhood category. H) Loadings for Principal Component 1. I) Expression of PD-L1 specifically on CD14+ monocytes and expression of IFNγ at large compared across clinical statuses (ANOVA). J) Logistic regression model of PD-1 expression probability on CD4+ T cells as a function of the local CD14+ monocyte abundance across clinical statuses (P < 0.01 for all main and interaction terms in the model).

The range of clinical statuses – biopsy, resection, and post-mortem – likely represents progressing disease. While other factors such as ongoing treatment and co-morbidities are surely important, they are not available as metadata. Nonetheless, we searched for patterns associated with putative severity. Although cellular abundances do not predict clinical status, SPACE also measures two metrics of cellular diversity. Alpha diversity, which measures the number and evenness of cell types in each FoV (Fig. 4a), is higher for biopsy and resection samples compared to postmortem (Fig 4b). Beta diversity, which measures the distinctiveness ME cellular compositions (Fig. 4c), is higher in biopsy samples, compared to either resection or post-mortem (Fig. 4d). Together, this suggests that early in disease progression, the distinctions among MEs blur as cell types intermix. Later, one or a few cell types dominate the granuloma, though not the same types in each patient.

Additionally, we searched for ensembles whose strength of spatial patterning correlates with clinical status. After narrowing our focus to cell types that were present in ≥80% of images, cisMI significantly associates with clinical status for one ensemble: CD4+ T cells, CD14+ monocytes, and TP macrophages. The strength of non-random patterning among these three cell types decreases as disease progresses (Fig. 4e). For more granular detail, we decomposed the census for each FoV into eight fractions: neighborhoods with: i) none of the three cell types, ii) just CD4+ T cells, iii) just CD14+ monocytes, iv) just TP Macs, v) CD4+ T cells and CD14+ monocytes, vi) CD4+ T cells and TP Macs, vii) CD14+ monocytes and TP Macs, and viii) all three cell types. We compared these fractions to fractions from randomized censuses simulated in SPACE. Thus, for each FoV, the eight fractions can be expressed as a Z-scores relative to null expectations. Extreme Z-scores indicate deviations from random patterning, such that average absolute Z-score correlates strongly with cisMI (Fig. 4f). Feeding the Z-scores for each FoV into PCA reveals that PC1 captures a large fraction of the variation (42.6%) and distinguishes biopsy from resection and post-mortem samples (Fig. 4g). PC1 loadings show that as disease progresses, neighborhoods with just 0–1 of the three cell types become less enriched, whereas neighborhoods with 2+ of the cell types become more enriched (Fig. 4h, stars). This agrees with the increase in cellular intermixing with more severe disease. However, one exception to this trend is the loss of CD4+ T cell colocalization with CD14+ monocytes (Fig. 4h, arrow).

To investigate functional interactions between CD4+ T cells and CD14+ monocytes in TB, we used 5 μm neighborhoods censused by SPACE to examine molecular expression. CD14+ monocytes expressed the inhibitory ligand PD-L1 at significantly higher levels in biopsy samples compared to resection or post-mortem (Fig. 4i). Although PD-L1 expression can be driven by Type 1 inflammatory signaling, little if any IFNγ was detected in the 30 FoVs (Fig. 4i). PD-L1 on CD14+ monocytes may suppress the activation of nearby T cells, preventing a robust inflammatory environment from forming. Using 20 μm neighborhoods, we modeled PD-1 expression probability on CD4+ T cells (a readout of antigen-specific activation) as a function of the frequency of nearby CD14+ monocytes. Nearby CD14+ monocytes strongly diminish CD4+ T cell activation in biopsy samples, weakly diminish it in resection samples, and exert no detectable effect in post-mortem samples (Fig. 4j). Thus, as disease progresses, CD14+ monocytes interact with CD4+ T cells less frequently, and these interactions become less suppressive when they do occur. Notably, these changes happen in a largely non-inflammatory signaling environment in which most other cell types are intermixing more rather than less. Therefore, it is possible that suppressive CD14+ monocyte interactions with CD4+ T cells help maintain the granuloma structure which contains pathogenic bacteria. If this structure decays without a corresponding increase in Type 1 inflammation, disease worsens. While such conjectures cannot be proven using observational data, they demonstrate that SPACE rapidly narrows a plethora of possible spatial patterns to a small set of statistically verified correlates of clinical status, streamlining the process of hypothesis generation.

## DISCUSSION

High-content spatial representations of biological tissues, including the images of human TB granulomas studied here, contain enormous amounts of information on tissue architecture and cellular arrangement. Existing analytical techniques – pairwise association metrics and MEs defined by clustering algorithms – only capture part of the insights available in such dense datasets. SPACE is, to the best of our knowledge, the first analysis platform to exhaustively quantify all possible spatial patterns. These patterns encompass any number of cell types or other entities and any degree of complexity, including context-dependent associations and quantitative gradients and orientations. Combined with the parsimonious assumption that higher-order patterns are built from lower-order patterns, SPACE ranks the exhaustively searched patterns, narrowing the enormous amount of information in the original data to a short list of candidate patterns most likely to reveal drivers of tissue organization.

As high-content spatial data on biological tissues becomes more sophisticated and prevalent, new innovations will be added to the SPACE platform. For example, SPACE currently operates on 2D image data, but 3D volumetric imaging promises to yield even more insights into complex tissue architecture [Li et al 2019]. While the information theoretic calculations behind cisMI are equally applicable, the program must be generalized to census 3D images. Similarly, while there is no theoretical limit to the number of ensembles for which cisMI can be calculated, there are practical limitations. For example, for a spatial transcriptomics data set with 1000 unique transcripts, exploring all ensembles including four mRNA species implies >4×10^10^ unique combinations, which would be computationally infeasible. Finally, SPACE analyses one image at a time, quantifying the difference between its true patterning vs. randomized expectations. Comparison across multiple images requires customized secondary analyses. As high-content spatial data becomes easier to collect in parallel for numerous samples, it will be more useful to directly quantify the spatial patterns that best distinguish sample groups from one another. To accomplish this in a single step, we are developing another information-theoretic metric, called transMI.

Even in its current form, the applications of SPACE are broad. Although we developed SPACE with high-content tissue images in mind, SPACE can census and analyze any properly formatted image-based data, from super-resolution microscopy of single cells to remote sensing of earth’s surface. The information-theoretic calculation of cisMI is agnostic to the source of the data, whether spatial or not. So long as randomized null expectations can be credibly simulated, SPACE essentially performs a nonparametric test for divergences between two distributions. Whereas common nonparametric tests (e.g., Wilcoxon Rank Sum, Kruskal-Wallis) only compare the first moment of 1-dimensional distributions, SPACE compares all moments of any-dimensional distributions, even when the underlying data is compositional. Thus, SPACE could prove to be an enormously useful statistical innovation. SPACE is available as a free, open-source R package that can be widely adopted and modified to support advanced analytical capabilities in a variety of scientific fields.

## METHODS

### Principles of SPACE

SPACE operates on a variety of image-based input data types, including images of quantitative molecular expression, segmented cells classified into phenotypes, and pixels classified into cellular phenotypes and/or acellular structures (Table 1, Fig 1a). SPACE can also accept tables of cellular XY centroids reporting the phenotype and/or average marker expression for each cell. Importantly, multiple inputs can be analyzed simultaneously, to integrate data at the molecular, cellular, and structural levels. Biological components at any of these levels are simply called “variables.” Across each layer of input data, thousands of randomly located circular neighborhoods are delimited (Fig 1b). While such sampled neighborhoods may overlap, no two neighborhoods are identically placed. The amount of each variable in each neighborhood is reported in a table called a “census” (Fig 1c–d). A census may include a variety of neighborhood sizes, for downstream analysis of patterning at different scales (e.g., adjacent cell contacts at 5–10μm vs. microanatomical zonation at 50–100μm). Inside each neighborhood, the contiguous patches of each variable are also recorded, respecting any truncation by the neighborhood’s boundary. By reassorting these patches into new hypothetical neighborhoods, randomized censuses are simulated (Fig. 1c). Randomized censuses represent null expectations in the absence of spatial patterning (Fig. 1d), while controlling for factors such cell size, morphology, and tissue geometry. At least 100 randomized censuses are simulated for downstream bootstrapping.

From the observed and randomized censuses, the degree of non-random patterning for any ensemble of variables can be quantified. The observed census gives the true distributions of heterotypic co-occurrence and homotypic occurrence for the included variables, while a randomized census gives the null expectations for these same distributions (Fig 1e). Although this is most easily depicted for an ensemble of two variables, it generalizes to ensembles of any size. The difference between these distributions is quantified using an analogue of mutual information called “cisMI,” measured in bits, which measures the degree of non-random patterning (Fig. 1f). Calculating cisMI requires several steps, to control for several important factors. First, the observed and two randomized censuses provide distributions of co-occurrence for the variables in the ensemble of interest, along with distributions for all subsets of these variables (Fig. S1a). These distributions are discretized with a user-specified number of bins per variable (Fig. S1b). More bins can resolve more intricate spatial patterns but also require more neighborhoods to be censused. The empirical bin probabilities are smoothed by calculating the Chao-Jost coverage estimator C [Chao & Jost 2012], normalizing the empirical distribution to C, and distributing the remaining 1-C probability mass uniformly across all possible but unobserved bins (Fig. S1c). This excludes bins representing impossible combinations of variables; for example, two cell types cannot sum to >100% of the area in a neighborhood. Then the Kullback-Leibler divergence is calculated between the observed and one randomized distribution, normalizing by the total number of possible bins to control for the compositional constraint on distribution shape. Per-bin Kullback-Leibler divergence between two randomized distributions is subtracted, to control for variation among randomized distributions that arises during stochastic simulation (Fig S1d). This yields an intermediate measure of cisMI. Because non-random patterning of single variables contributes to apparent non-random patterning of 2-variable ensembles (and non-random patterning of 2-variable ensembles contributes to apparent non-random patterning of 3-variable ensembles, and so on), the intermediate measure for all lower-order subsets is subtracted from higher-order ensembles, giving a final measure of cisMI (Fig. S1e). This regularizes cisMI for higher-order ensembles, invoking a parsimonious assumption that spatial patterning is driven by the simplest (lowest-order) interactions. Remaining cisMI for higher-order ensembles reflects patterning that cannot be explained purely by lower-order terms. CisMI is measured repeatedly using many simulations of randomized distributions, to estimate uncertainty via bootstrapping (Fig S1f). If ≥95% of cisMI estimates exceed 0, then the ensemble displays a significantly non-random spatial pattern. A single ensemble can be queried for hypothesis testing, or these calculations can be looped over many ensembles to explore many possible spatial patterns, where ensembles are ranked from strongest to weakest patterning (highest to lowest cisMI, Fig 1g).

Importantly, a high cisMI score indicates strong non-random patterning, but it does not indicate the type of pattern, whether co-occurrence, mutual exclusion, or non-linear. Thus, an ensemble of interest with significant cisMI can be selected for further exploration. From the observed census, the distribution of heterotypic co-occurrence is generated in V-dimensional space (where V is the number of variables in the ensemble), and a self-organizing map places a curve through the areas of highest data density (Fig. 1h). The curve is then projected into lower dimension by plotting the value of each variable along the curve separately. The resulting “covariation plot” shows how each variable in the ensemble covaries as the tissue is traversed along a hidden but illustrative (i.e., latent) path (Fig 1i). This reveals context-dependent associations, quantitative gradients, and other features of the ensemble’s spatial patterning. To visualize this in the original tissue context, manual gates can be drawn on the covariation plot, splitting the image into regions with characteristic amounts or gradients of each of the involved variables (Fig 1i). These regions are mapped back onto the original image with false coloring for easy visualization (Fig 1j). Because mapped regions are identical in format to segmentation masks and pixel-classified images, they can be recycled as inputs for another round of SPACE analysis, to find hierarchical spatial relationships among the regions themselves (Fig 1j).

### Implementation and Availability of SPACE

SPACE is written in the R computing language and is freely available as an open-source R package on Github [link page when ready], where code documentation and a tutorial are also available. All other analyses in this paper are performed in R as described in the text and figure legends. Data and code required for all analyses in this paper are freely available on Github [link page when ready].

## Supplementary Material

Supplement 1

Supplement 2

Supplement 3

Supplement 4

## Figures and Tables

**Table 1.** Comparison of SPACE with existing spatial analysis platforms. “Input Data” refers to the actual data on which statistical calculations are performed. “Pattern Detection” refers to patterns that are systematically explored and reported.

Program Details		Input Data					Pattern Detection				
Name	Platform	Full Images			Centroid Tables		Plexity		Complexity		Microenvironments
		Expression	Objects	Pixels	Expression	Objects	1–2	3+	Linear	Nonlinear	Categorical
SPACE	R	yes	yes	yes	yes	yes	yes	yes	yes	yes	yes
CODEX Toolkit	GUI	no	no	no	yes	yes	yes	no	yes	no	yes
CytoMAP	Matlab	no	no	no	yes	yes	yes	no	yes	no	yes
Giotto	R	no	no	no	yes	yes	yes	no	yes	no	yes
HistoCAT	GUI	no	no	no	yes	yes	yes	no	yes	no	no
ImaCytE	GUI	no	no	no	no	yes	yes	no	yes	no	no
SIMPLI	Nextflow	no	no	no	yes	yes	yes	no	yes	no	no
SPIAT	R	no	no	no	yes	yes	yes	no	yes	no	yes
Visinity	GUI	no	no	no	no	yes	yes	no	yes	no	yes

## Data Availability

All data used in this study are publicly available from their original studies: mouse data are available in [Radtke et al 2020] and human data are available in [McCaffrey et al 2022]. The SPACE R package and documentation is currently available by request only, but it will be released freely and publicly on Github after peer review and publication, along with the complete R code used for all analyses in this study.
