# Supplementary material for: Spatial Patterning Analysis of Cellular Ensembles (SPACE) discovers complex spatial organization at the cell and tissue levels
Source: bioRxiv. 2024 May 30:2023.12.08.570837. Originally published 2023 Dec 10. Preprint. [Version 2] doi: 10.1101/2023.12.08.570837 (PMC10760187; doi:10.1101/2023.12.08.570837)
Supplement: Supplement 4 [file NIHPP2023.12.08.570837v2-supplement-4.pdf]

bioRxiv preprint doi: https://doi.org/10.1101/2023.12.08.570837; this version posted May 30, 2024. The copyright holder for this preprint (which was not certified by peer review) is the author/funder. This article is a US Government work. It is not subject to copyright under 17 USC 105 and is also made available for use under a CC0 license.

Supplementary Figure and Table Legends

Table S1. Lineage marker expression expectations used for semi-supervised cellular phenotyping in the mouse LN. 1 indicates expected expression, 0 indicates expected lack of expression, and NA indicates no expectation.

Table S2. Centroid table of cellular positioning and phenotype in the mouse LN.

Table S3. Key features of ME compositions required to demonstrate patterns of the mouse LN organization discovered by SPACE.

Figure S1. Cellular segmentation and semi-supervised phenotyping for the mouse LN. A) The original 42-plex IBEX image of a mouse popliteal LN. Six channels are shown: CD3 (green), B220 (blue), F480 (pink), DEC205 (red), CD21 (yellow), αSMA (white). B) Segmentation mask of single cells, generated in Cellpose 2.0 on default settings with JOJO as nucleus and CD45 as membrane. C) For each of 20 lineage markers in the reduced panel, the mean fluorescence intensity (MFI) across cells forms a distribution, for which a binarization threshold is chosen using the IsoData algorithm. CD3 is shown as an example. D) MFI is transformed into a probability-of-expression using a Hill function sigmoid curve with EC50 equal to the marker's binarization threshold and Hill exponent equal to 4. E) The distribution of MFI across cells is transformed into a distribution of probability-of-expression, in which positive and negative cells are more clearly separated. F) For each cell, the expression probability is calculated for each marker. Cell 500 out of 17,932 is shown as an example. This expression profile is compared to the canonical profile for each expected cell type. T-reg and NK are shown as examples. In each comparison, the focal cell's expression probabilities are unchanged for markers expected to be positive and subtracted from 1 for markers expected to be negative. The geometric mean of these values gives a probability score describing how likely the focal cell is to belong to the canonical type. Expression probabilities are omitted from the geometric mean when they are uninformative; for example, NK expression of CD11b is variable, so CD11b is uninformative when evaluating whether a cell belongs to the NK type. G) For each cell in the data set, a probability score is calculated for each canonical cell type, and the maximum score indicates the most likely type. H) The maximum score is close to 1 for most cells, indicating a good match. 97.2% of cells have a maximum score > 0.5 and are categorized into one of the canonical types. 2.8% of cells have a maximum score ≤ 0.5. Here, such cells are considered "unknown;' however, unsupervised clustering approaches can be applied to this subset of cells to discover unexpected types.

Figure S2. Comparison of cisMI Z scores measured by SPACE for all ensembles of up to 3 out of the 19 cell types in the mouse LN from the segmentation image vs. the centroid table, using a Gaussian linear model.

Figure S3. Volumetric image of a mouse LN to visualize different slicing angles. A) The full 3d volume viewed from a mid-sagittal angle shows the inside-out gradient of CD8 to CD4 T cells. B) A single 2d slice viewed from a mid-sagittal angle also shows the inside-out gradient of CD8 to CD4 T cells. C) The full 3d volume viewed slightly oblique to a mid-sagittal angle shows a right-to-left gradient of CD8 to CD4 T cells. D) A single 2d slice slightly oblique to a mid-sagittal angle also shows a right-to-left gradient of CD8 to CD4 T cells. White arrow show where the CD4 T cells that separate the CD8 T cells from the B follicle disappear fully on the right, but not fully on the left.

bioRxiv preprint doi: https://doi.org/10.1101/2023.12.08.570837; this version posted May 30, 2024. The copyright holder for this preprint (which was not certified by peer review) is the author/funder. This article is a US Government work. It is not subject to copyright under 17 USC 105 and is also made available for use under a CC0 license.

Figure S4. All K-means clustering runs to define MEs on the mouse LN across K values from 2 to 25. A) Specific key features recovered by each clustering run. B) Total key features recovered by each clustering run.

Figure S5. Pairwise correlations of transcript abundance across the 2660 spatial spots in the human intestinal cancer sample. A) Comparison of standardized counts of GPX2 and MGAM2. B) Comparison of standardized counts of MGAM2 and PPP1R1B. C) Comparison of standardized counts of PPP1R1B and GPX2. D) Comparison of standardized counts of IGHG4 and SFRP2. All comparisons were made with a Gaussian linear model.

https://doi.org/10.1101/2023.12.08.570837 ... the copyright holder for this preprint ... not subject to copyright under 17 USC ... CC0 license.

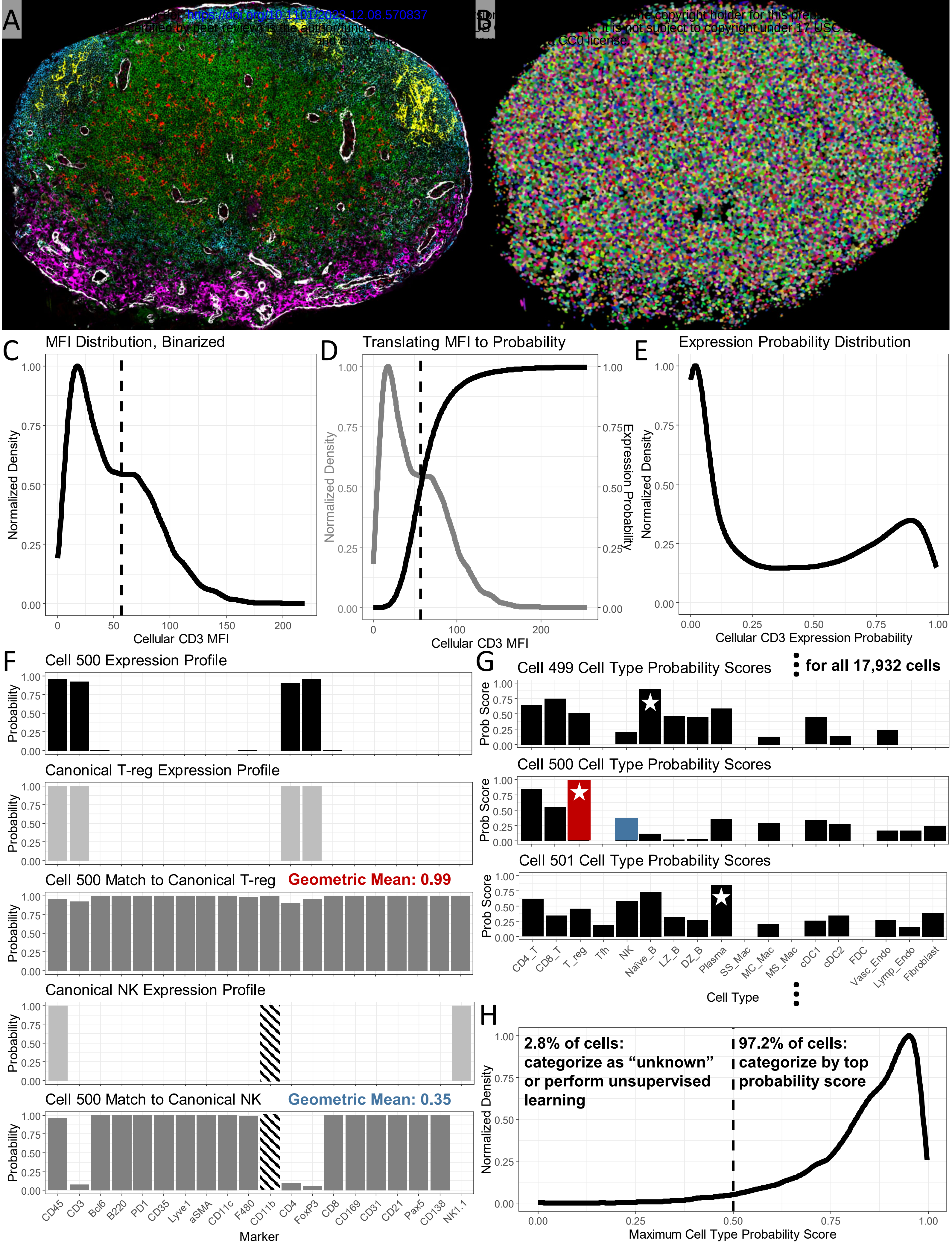

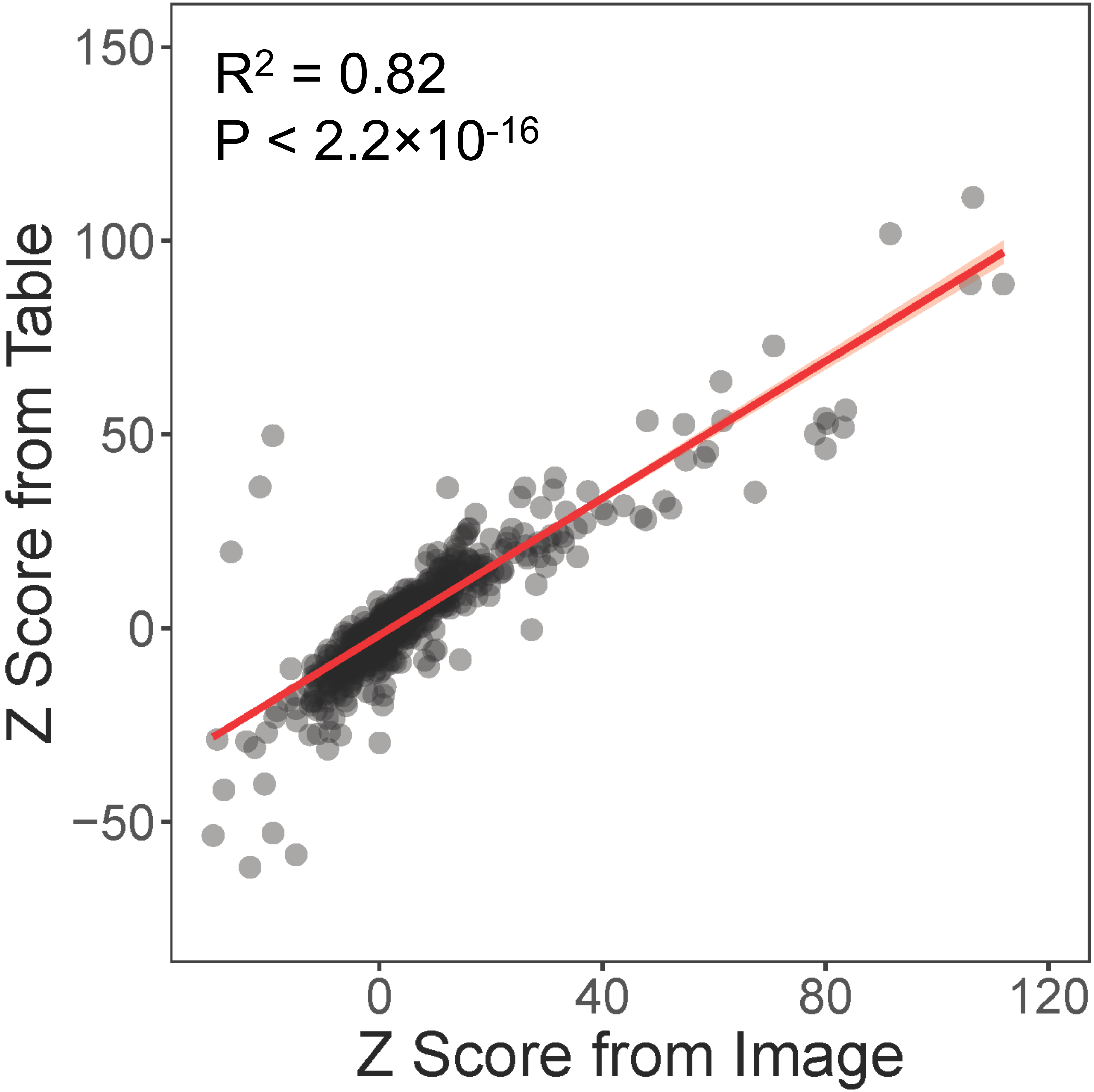

bioRxiv preprint doi: https://doi.org/10.1101/2023.12.08.570837; this version posted May 30, 2024. The copyright holder for this preprint (which was not certified by peer review) is the author/funder. This article is a US Government work. It is not subject to copyright under 17 USC 105 and is also made available for use under a CC0 license.

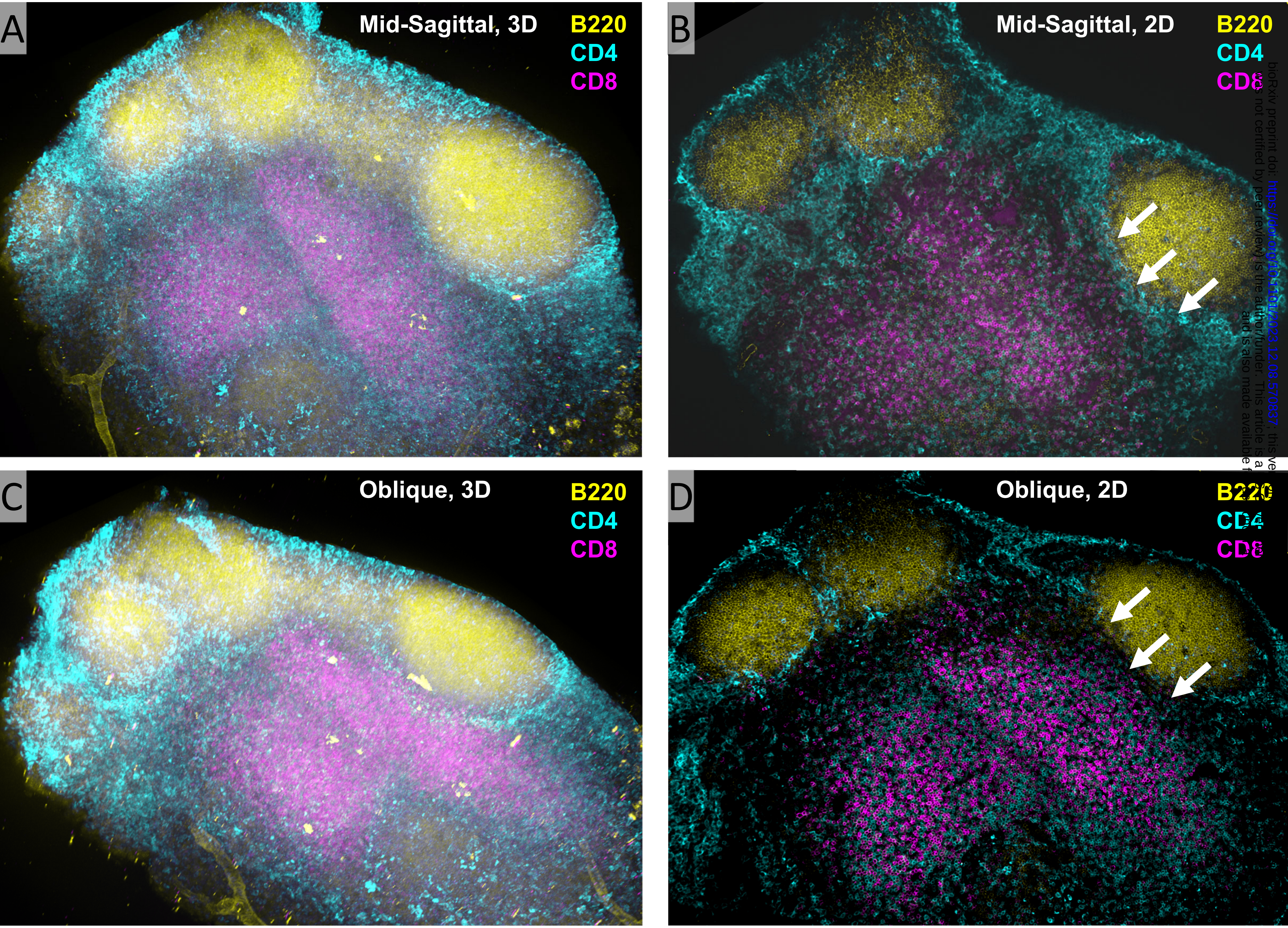

bioRxiv preprint doi: https://doi.org/10.1101/2023.12.08.570837; this ve
is not certified by peer review) is the author/funder. This article is a
and is also made available f

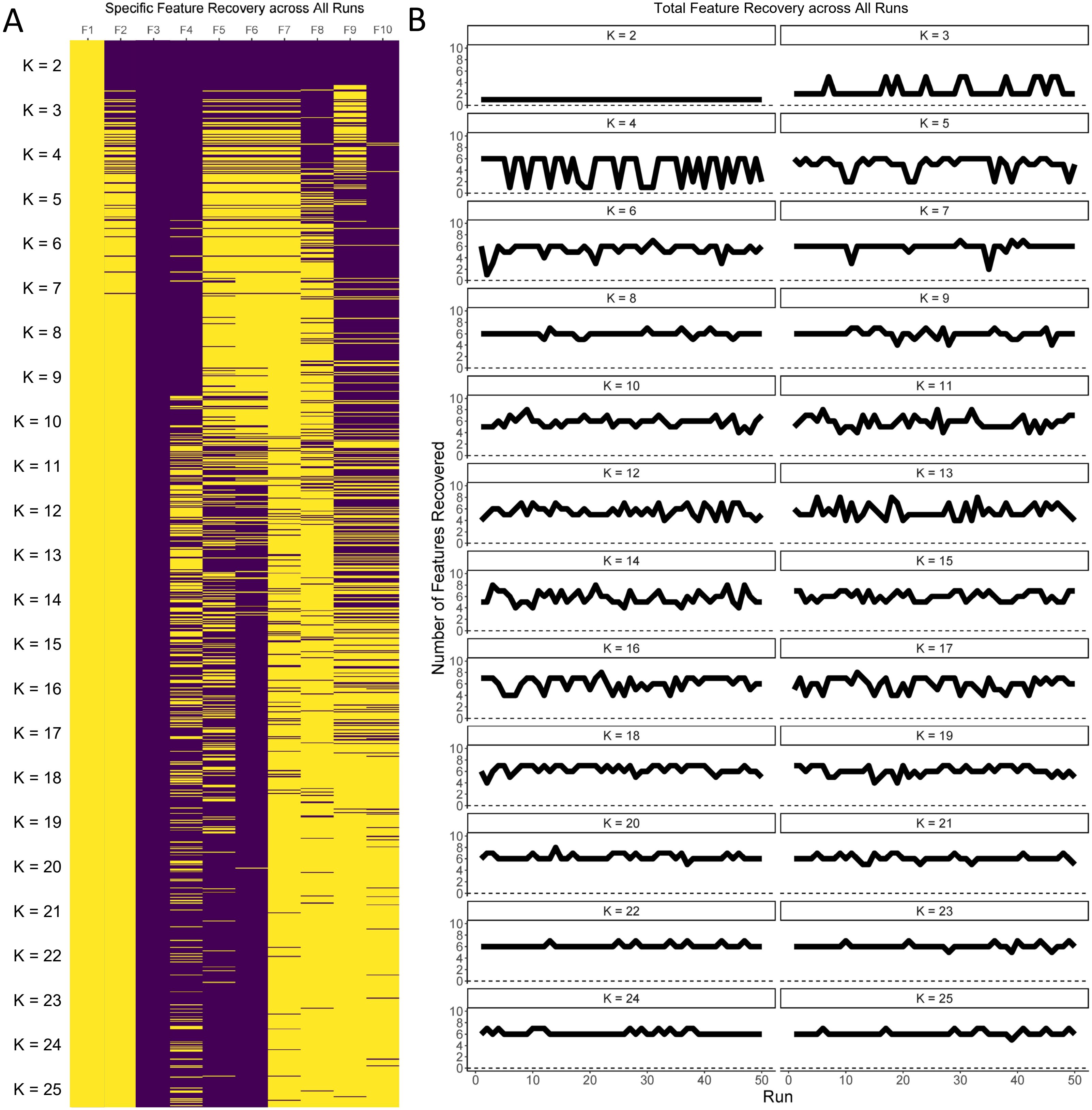

bioRxiv preprint doi: https://doi.org/10.1101/2023.12.08.570837; this version posted May 30, 2024. The copyright holder for this preprint (which was not certified by peer review) is the author/funder. This article is a US Government work. It is not subject to copyright under 17 USC 105 and is also made available for use under a CC0 license.

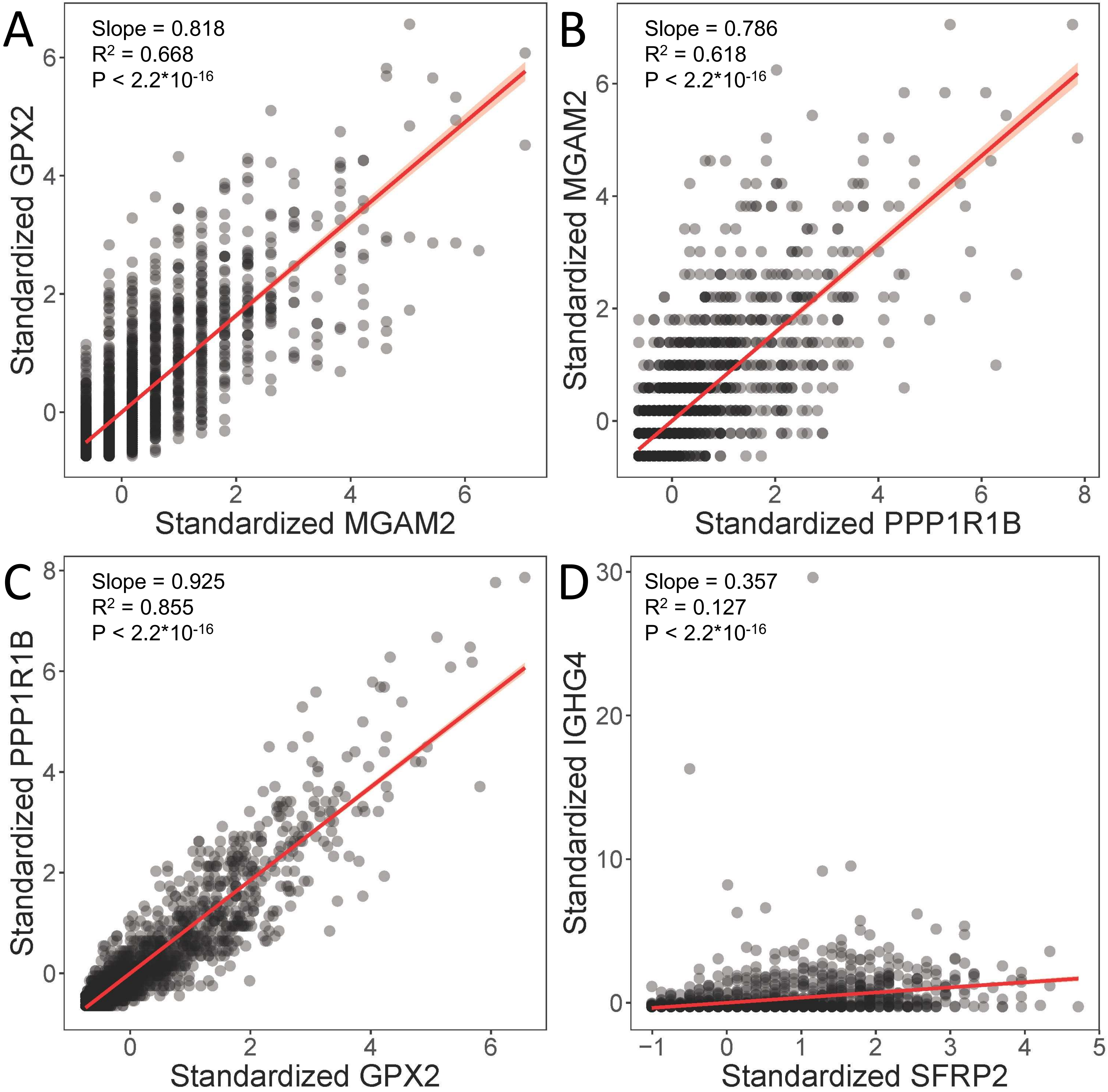
A
Slope = 0.818
R2 = 0.668
P < 2.2*10-16
Standardized GPX2
Standardized MGAM2
B
Slope = 0.786
R2 = 0.618
P < 2.2*10-16
Standardized MGAM2
Standardized PPP1R1B
C
Slope = 0.925
R2 = 0.855
P < 2.2*10-16
Standardized PPP1R1B
Standardized GPX2
D
Slope = 0.357
R2 = 0.127
P < 2.2*10-16
Standardized IGHG4
Standardized SFRP2
