# Supplementary material for: Spatial Patterning Analysis of Cellular Ensembles (SPACE) enables statistically robust discovery of complex spatial organization at the cell and tissue level
Source: bioRxiv. 2023 Dec 10:2023.12.08.570837. Preprint. [Version 1] doi: 10.1101/2023.12.08.570837 (PMC10760187; doi:10.1101/2023.12.08.570837)
Supplement: Supplementary file 1 [file media-1.pdf]

Text S1

Instead of a full image of segmented cells color-coded by the cell types (Fig. 2b), SPACE can alternatively be applied to a table of cell centroid coordinates (Table S2). In this case, the cisMI values agree very well with the image-based values (Fig. S3a). However, the table-based cisMI values are systematically lower (Fig. S3b), reflecting the enhanced statistical power that SPACE achieves by using full images as input data. Ensembles whose cisMI is reduced the most when using centroid tables typically include cell types that are relatively rare in the tissue (Fig S3c). Thus, table-based analyses are valid but less powerful than SPACE's default image mode.

Text S2

We also applied existing spatial analysis approaches to the same mouse LN to search for the patterns highlighted by SPACE. In accordance with existing approaches, we used the table of centroids and phenotypes for segmented cells (Table S2), rather than the full image of segmented cells (Fig. 2b). We censused each centroid's neighbors within 20 μm using the true phenotypes as well as permuted phenotypes to describe null expectations in the absence of spatial patterning (Fig S5a).

First, we examined pairwise associations among cell types. Specifically, we counted how often each pair of cell types were neighbors, and we compared these counts to randomized expectations from permutations of cellular phenotypes (Fig. S5b). CD4+ T cells, CD8+ T cells, naïve B cells, LZ B cells, and DZ B cells all have significantly more homotypic neighbors than expected by random chance (Fig. S5c, gray boxes), reflecting their assortment into distinct tissue zones. CD4+ and CD8+ T cells neighbor one another more frequently than expected by random chance, and they each neighbor naïve B cells less frequently than expected by random chance (Fig. S5c, white boxes). This reflects the coexistence of CD4+ and CD8+ T cells in the T zone separate from B follicles, but it fails to suggest that CD4+ and CD8+ T cells form opposing gradients within the T zone. This is because these opposing gradients are context-specific to the T zone and contradict the tissue-wide co-occurrence pattern. Only the tissue-wide pattern is accessible to pairwise analyses.

Similarly, both LZ and DZ GC B cells neighbor naïve B cells more frequently than expected (Fig. S5c, black boxes), reflecting the embedding of GCs in naïve B follicles. However, there is no indication that DZs preferentially face the interior of naïve B follicles compared to LZs, because this requires information on all three of the involved cell types simultaneously. Again, multi-partner patterns are inaccessible to pairwise analyses.

To escape the constraints of pairwise analyses, we used machine learning to define MEs of consistent cell type compositions (Fig. S5d). While we used K-means, any clustering or topic modeling algorithms yields similar information: the mean cellular composition of each ME and a visual map of which cells belong to each ME (Fig. S5e). For a representative clustering using K=15 MEs, the cellular compositions (Fig. S5f) and the visual map (Fig. S5g) are shown. While discrete MEs cannot capture smooth cellular gradients in a strict sense, we translated the gradients and orientations identified by SPACE into 10 key features that could be suggested by discrete MEs in principle (Table S3). For example, if the ME(s) enriched for CD4+ T cells are also enriched for CD8+ T cells, then they likely represent the T cell zone (Fig. S5f, MEs 1-6). Within these MEs, if CD4+ T cell content is negatively correlated with CD8+ T cell content, one might argue that the MEs demonstrate opposing gradients (Fig. S5f, MEs 1-6). But other key features may be missing; for example, if LZ GC B cells and DZ GC B cells are most enriched in the same ME (Fig. S5f, ME 12), then the MEs do not distinguish LZ vs. DZ and therefore cannot indicate GC orientation. Across 50 K-means clustering runs with K=15, we found that different runs capture different key features, but no single run captures more than 8 of the 10 key features (Fig. S5h).

A priori, there is no reason to seek exactly 15 MEs, so we varied K from 2 to 25 and performed 50 runs at each value. The average silhouette score, which measures global clustering quality to guide the choice of K, peaks at 3 (Fig. S5i, black line): from a purely computational perspective, it is most

parsimonious to define only 3 MEs. However, when K=3, only 8.2% of key features are recovered on average. Recovery of key features peaks at K=15, but still only 57.0% of these features are recovered on average (Fig. S5i, red line), and the silhouette score is far from optimal. Different key features tend to be recovered at different K values (Fig. S6a), but no single clustering run at any K value recovered more than 8 of 10 (Fig. S6b). Because the full exploratory output of SPACE (Fig. 2c) captures many more patterns than just the gradients and orientations discussed here, the true success rate of MEs may be substantially lower. Although machine learning of cellular MEs detects general trends of co-assortment, it does not reliably detect more complex features of cellular patterning, such as context-dependent gradients and orientations.

Text S3

The original study reports some patterns of cell type- and/or ME-specific expression of functional markers, which were discovered using customized analyses. Because SPACE naturally operates on multiple layers of image-based data simultaneously (Fig. 1a), we were able to search for these patterns automatedly. For example, it was reported that T-regs express Ki67 more frequently than conventional CD4+ T cells, CD8+ T cells, or B cells. To evaluate this cellular context-specific expression in SPACE, we censused across both the cell phenotype maps and the raw Ki67 signal, using a 5 µm radius to approximate a single-cell spatial scale. Tripartite cisMI among Ki67 and two different lymphocyte types is higher when one of the two lymphocyte types is T-reg, indicating that T-regs have a distinct expression frequency of Ki67 (Fig. S7a, black box). Further characterization agreed with the original study: our 5 µm neighborhoods show that T-regs express Ki67 more frequently than other lymphocyte types. (Fig. S7b).

Because SPACE detects any non-random spatial pattern, it is also informative to find ensembles that do not have significant cisMI, indicating that their patterning is effectively random. For example, the original study reported ME-specific expression of IDO1 by CD11c+ DCs. In particular, IDO1 expression by CD11c+ DCs exceeds baseline in the Intermediate Monocyte (IntMono), Fibrosis (Fib), and Mcore1 MEs and is below baseline in the Lymphocyte Cuff (Lcuff) and Myeloid Core 2 (Mcore2) MEs. This suggests that CD11c+ DCs respond to their local ME by modulating IDO1 expression. In this case, SPACE ought to measure significant tripartite cisMI among CD11c+ DCs, IDO1, and any of these five MEs, but it does not (Fig. S7c, gray box). Yet pairwise cisMI between IDO1 and any of these five MEs is consistently positive (Fig. S7c, black box). Together, this suggests that MEs have different levels of IDO1 expression at large, but it is not driven by CD11c+ DCs sensing and responding to their surroundings. If the local ME does not cause CD11c+ DCs to change their IDO1 expression, an alternative explanation for the originally observed pattern is that some unknown factor causes both ME content and IDO1 expression to covary globally across FoVs (Fig. S7d). For example, if IntMono-rich FoVs are also IDO1-rich, then a CD11c+ DC found in such an FoV would likely belong to the IntMono ME and be IDO1+, and the converse for IntMono-poor and IDO1-poor FoVs. In support of this explanation, total IDO1 expression correlates positively with total IntMono content across FoVs (Fig. S7e). By controlling for lower-order patterns (e.g., IDO1 expression at large across MEs), SPACE discerns that some apparent higher-order patterns (e.g., IDO1 expression specific to CD11c+ DCs across MEs) are actually not significant. Prioritizing lower-order patterns reflects an assumption of parsimony that allows SPACE to infer the most likely drivers of spatial patterns.

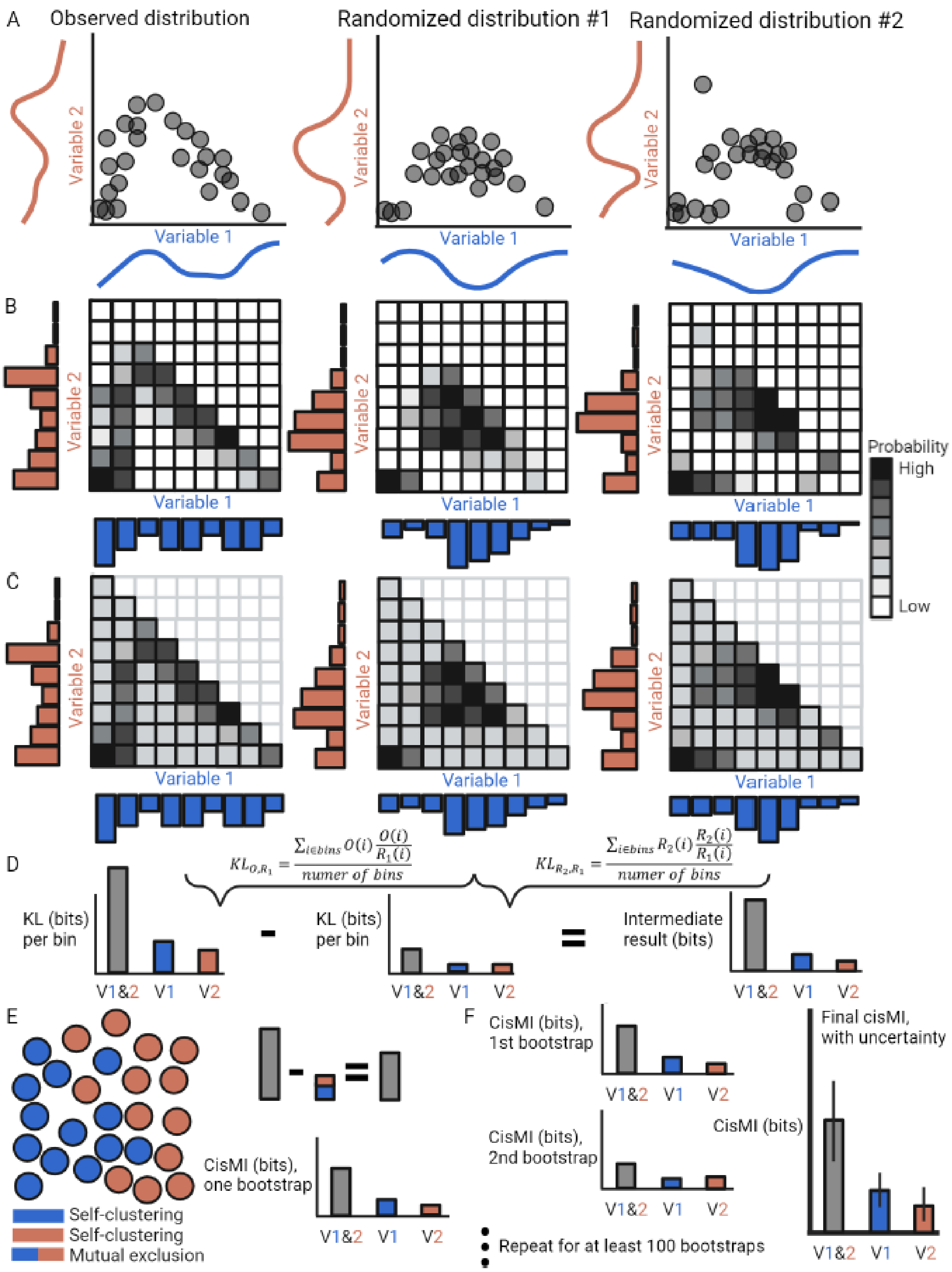

Figure S1. Steps to calculate cisMI. A) The observed and two randomized distributions of heterotypic co-occurrence and homotypic occurrence for two variables. While this illustration considers a 2-variable ensemble, ensembles including any

number of variables are possible. B) The distributions are discretized, in this case using 9 bins per variable. Empirical bin probabilities are given by the fraction of censused neighborhoods in each bin. C) The empirical distributions are smoothed by discarding bins that represent impossible combinations of variables (summing to > 100%) and assigning a small probability to all bins that are possible but unobserved. D) For the focal ensemble and all subsets, Kullback-Leibler divergence is calculated and normalized by the total number of bins, between the observed and one randomized census, as well as between two randomized censuses. The latter is subtracted from the former. E) Lower-order patterns contribute to the appearance of higher-order patterns; for example, two cell types that independently self-cluster as 1-variable patterns also contribute to the appearance of mutual exclusion, a 2-variable pattern. Thus, cisMI for lower-order subsets is subtracted from higher-order ensembles. F) These calculations are repeated using many pairs of simulated randomized distributions, yielding bootstrap cisMI estimates to quantify uncertainty. Created with Biorender.com.

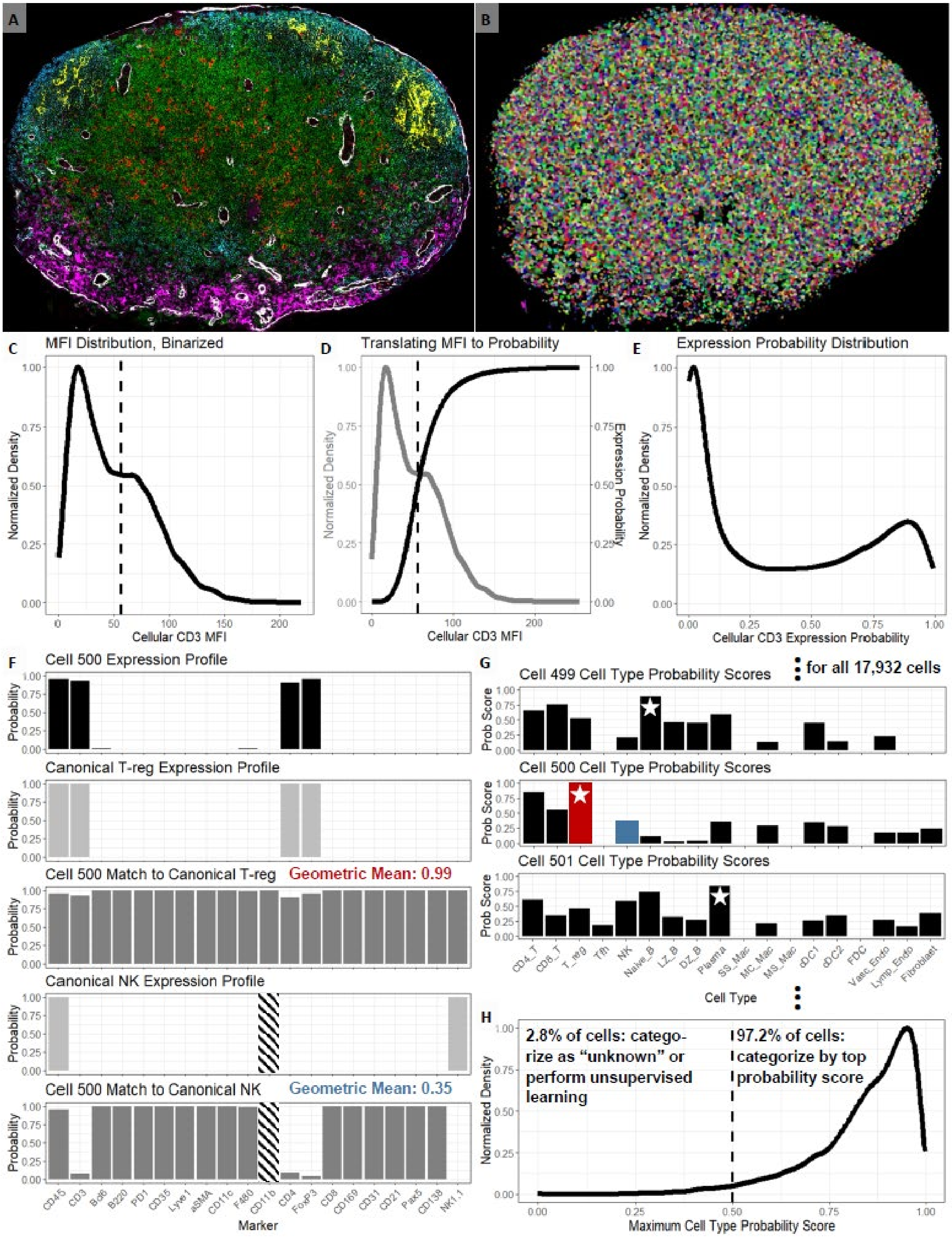

Figure S2. Cellular segmentation and semi-supervised phenotyping. A) The original 42-plex IBEX image of a mouse popliteal LN. Six channels are shown: CD3 (green), B220 (blue), F480 (pink), DEC205 (red), CD21 (yellow), αSMA (white). B) Segmentation mask of single cells, generated in Cellpose 2.0 on default settings with JOJO as nucleus and CD45 as membrane. C) For each of

20 lineage markers in the reduced panel, the mean fluorescence intensity (MFI) across cells forms a distribution, for which a binarization threshold is chosen using the IsoData algorithm. CD3 is shown as an example. D) MFI is transformed into a probability-of-expression using a Hill function sigmoid curve with EC_{50} equal to the marker's binarization threshold and Hill exponent equal to 4. E) The distribution of MFI across cells is transformed into a distribution of probability-of-expression, in which positive and negative cells are more clearly separated. F) For each cell, the expression probability is calculated for each marker. Cell 500 out of 17,932 is shown as an example. This expression profile is compared to the canonical profile for each expected cell type. T-reg and NK are shown as examples. In each comparison, the focal cell's expression probabilities are unchanged for markers expected to be positive and subtracted from 1 for markers expected to be negative. The geometric mean of these values gives a probability score describing how likely the focal cell is to belong to the canonical type. Expression probabilities are omitted from the geometric mean when they are uninformative; for example, NK expression of CD11b is variable, so CD11b is uninformative when evaluating whether a cell belongs to the NK type. G) For each cell in the data set, a probability score is calculated for each canonical cell type, and the maximum score indicates the most likely type. H) The maximum score is close to 1 for most cells, indicating a good match. 97.2% of cells have a maximum score > 0.5 and are categorized into one of the canonical types. 2.8% of cells have a maximum score ≤ 0.5. Here, such cells are considered "unknown;' however, unsupervised clustering approaches can be applied to this subset of cells to discover unexpected types.

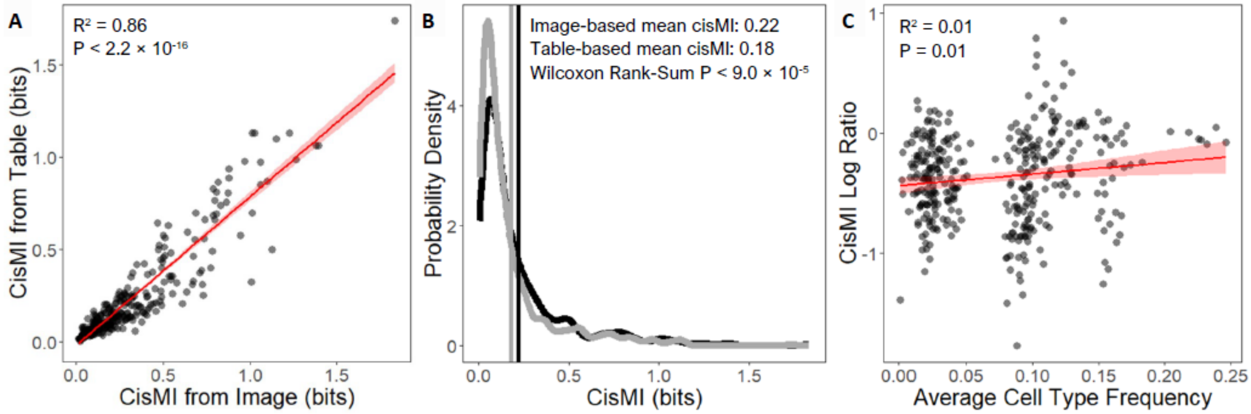

Figure S3. Comparison of SPACE performed on segmentation image vs. centroid table for 19 cell types in the mouse LN. A) Image- vs. table-based cisMI values correlate strongly across all significant ensembles discovered by SPACE. B) Table-based cisMI is lower than image-based cisMI, on average. C) The log-ratio of table-based to image-based cisMI for an ensemble is correlated with the average frequency of the involved cell types. Table-based cisMI is lower relative to image-based for ensembles which contain rarer cell types.

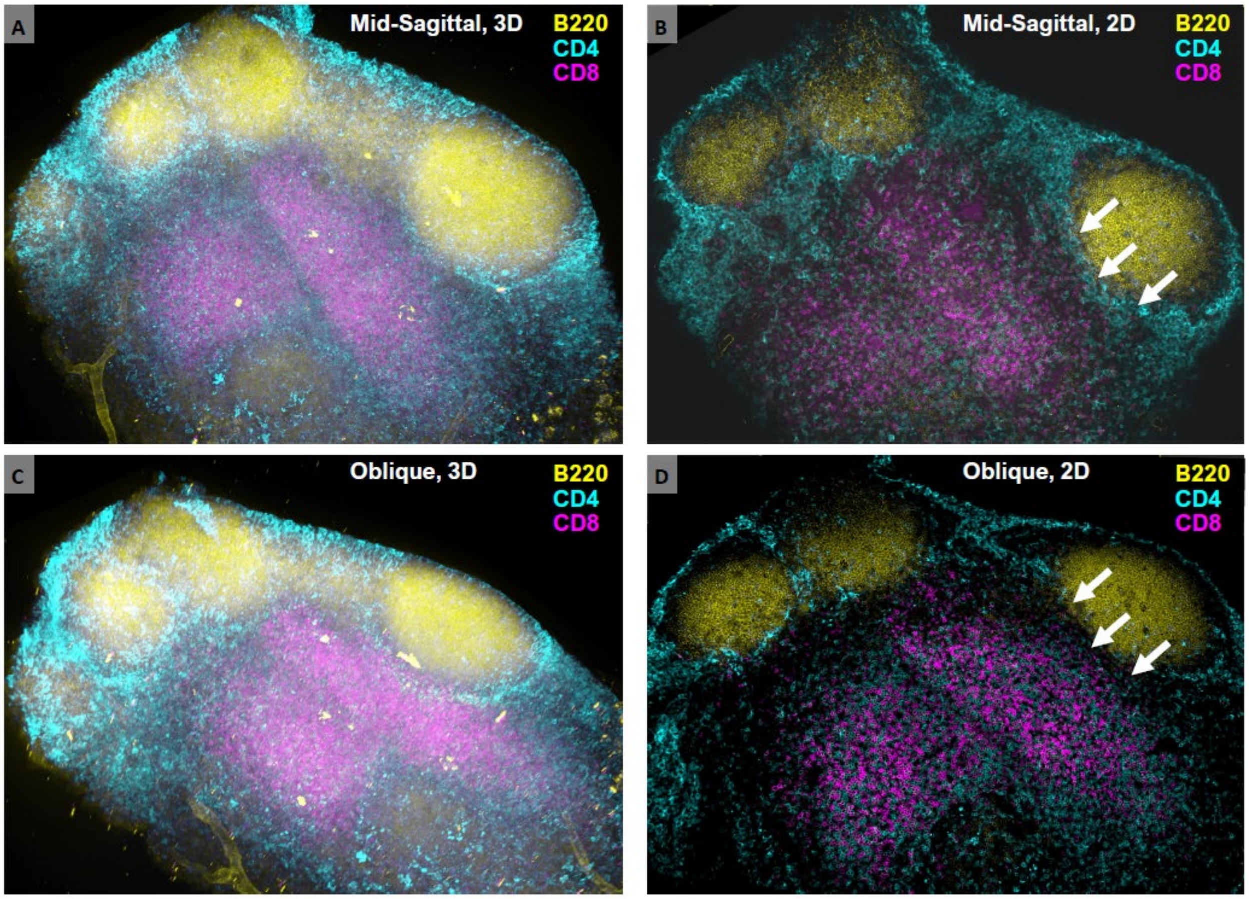

Figure S4. Volumetric image of a mouse LN to visualize different slicing angles. A) The full 3d volume viewed from a mid-sagittal angle shows the inside-out gradient of CD8+ to CD4+ T cells. B) A single 2d slice viewed from a mid-sagittal angle also shows the inside-out gradient of CD8+ to CD4+ T cells. C) The full 3d volume viewed slightly oblique to a mid-sagittal angle shows a right-to-left gradient of CD8+ to CD4+ T cells. D) A single 2d slice slightly oblique to a mid-sagittal angle also shows a right-to-left gradient of CD8+ to CD4+ T cells. Specifically, the CD4+ T cells that separate the CD8+ T cells from the B follicle disappear fully on the right (white arrows) but not fully on the left.

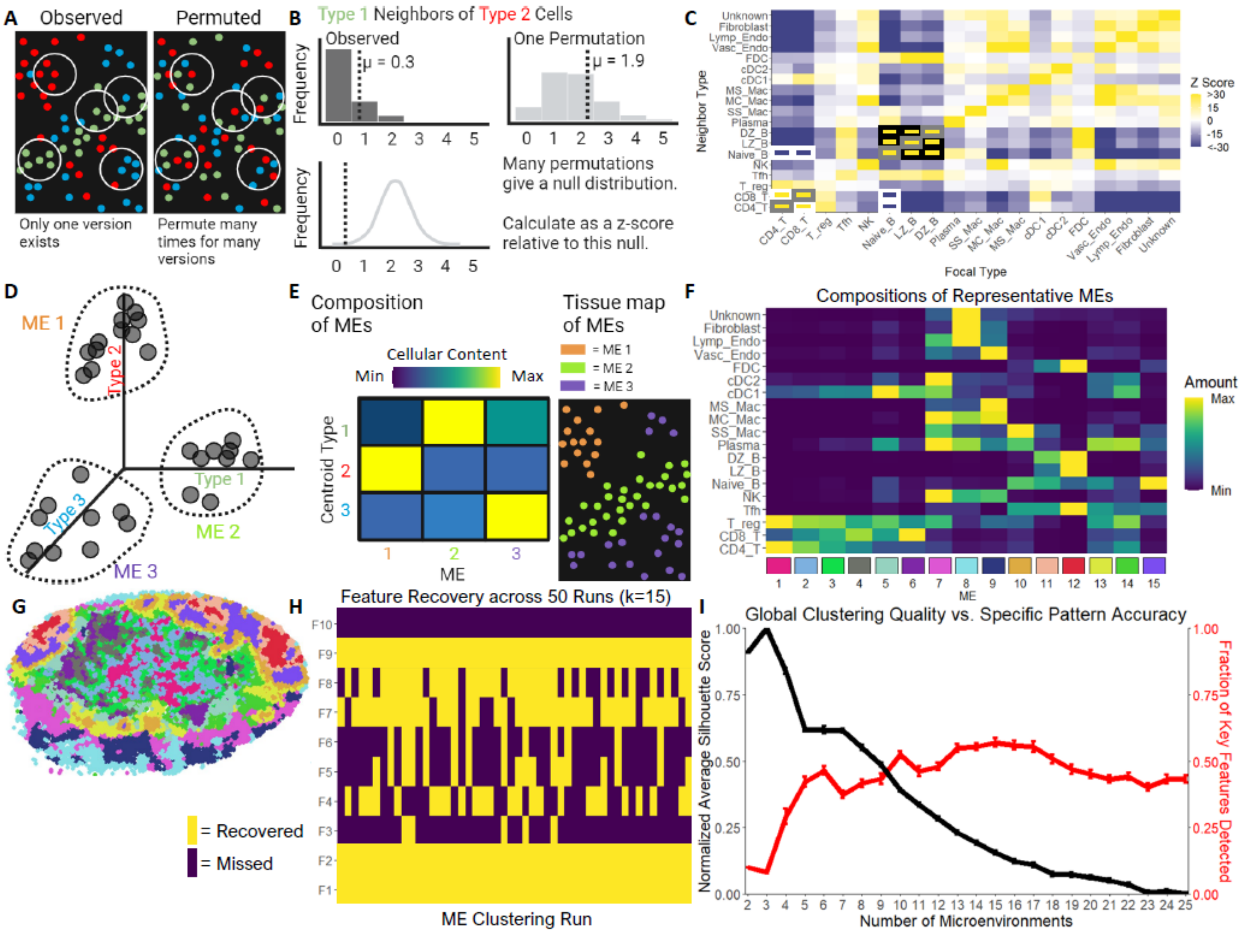

Figure S5. Application of existing techniques to patterns identified by SPACE. A) Schematic of collection of observed and randomized censuses from centroid tables. B) Schematic of pairwise analysis comparing neighbor frequency to null expectations. C) Pairwise analysis of the frequency with which each focal cell type has the indicated neighbor. Z-scores are relative to 100 random permutations of cellular phenotypes. D) Schematic of ME definition via clustering-based machine learning. E) Schematic of ME composition and visual mapping. F) ME compositions from a representative clustering seeking K=15 MEs on the centroid table from the mouse lymph node. G) ME visual mapping from the representative clustering. H) Key feature recovery for each of 50 clustering runs with K=15. I) Silhouette scores for the quality of clustering (black) and the fraction of key features recovered (red) for repeated clustering runs across a range of K values. Created with Biorender.com (Panels A, B, D, E).

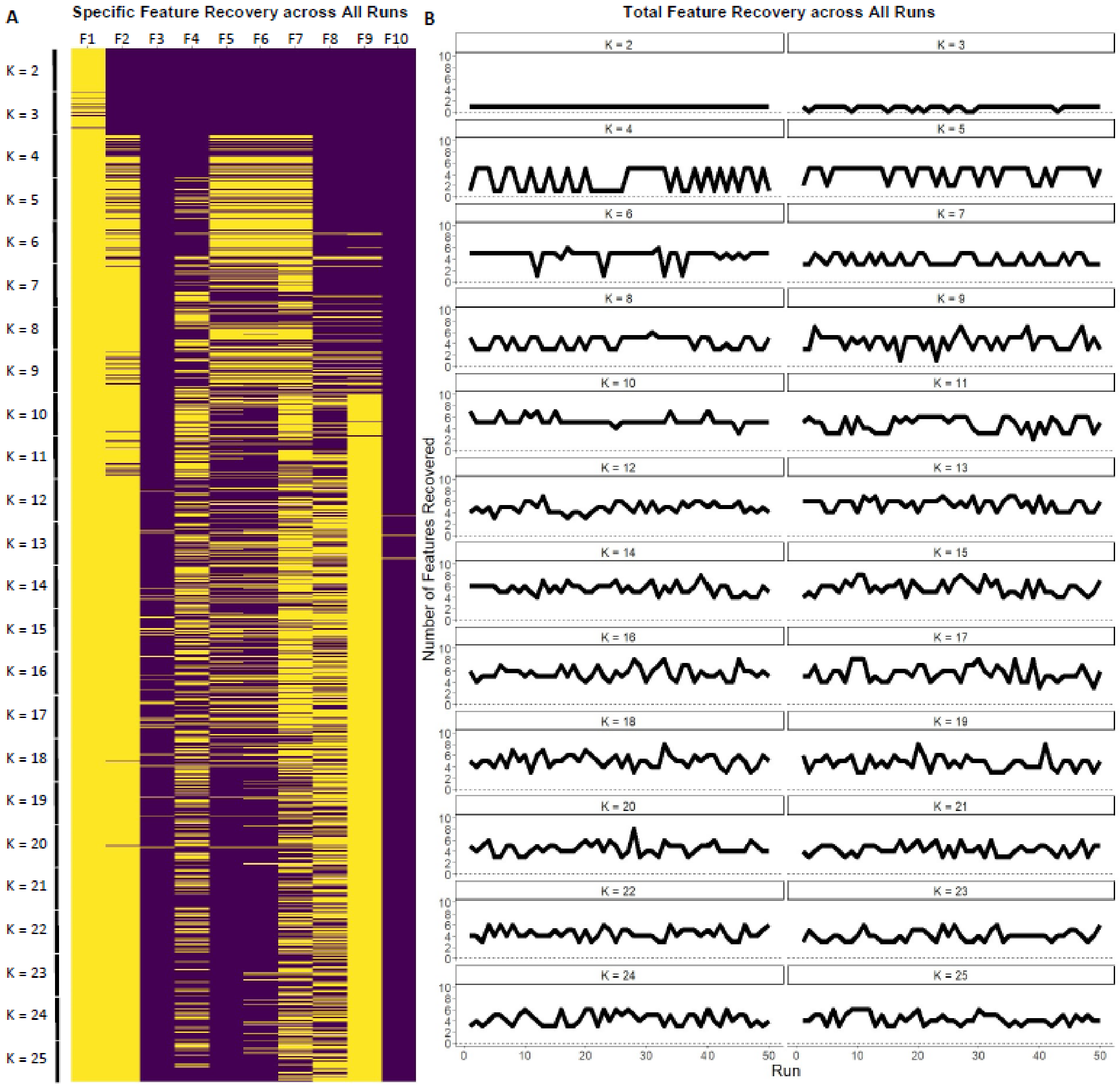

Figure S6. All K-means clustering runs to define MEs across all K values from 2 to 25. A) Specific key features recovered by each clustering run. B) Total key features recovered by each clustering run.

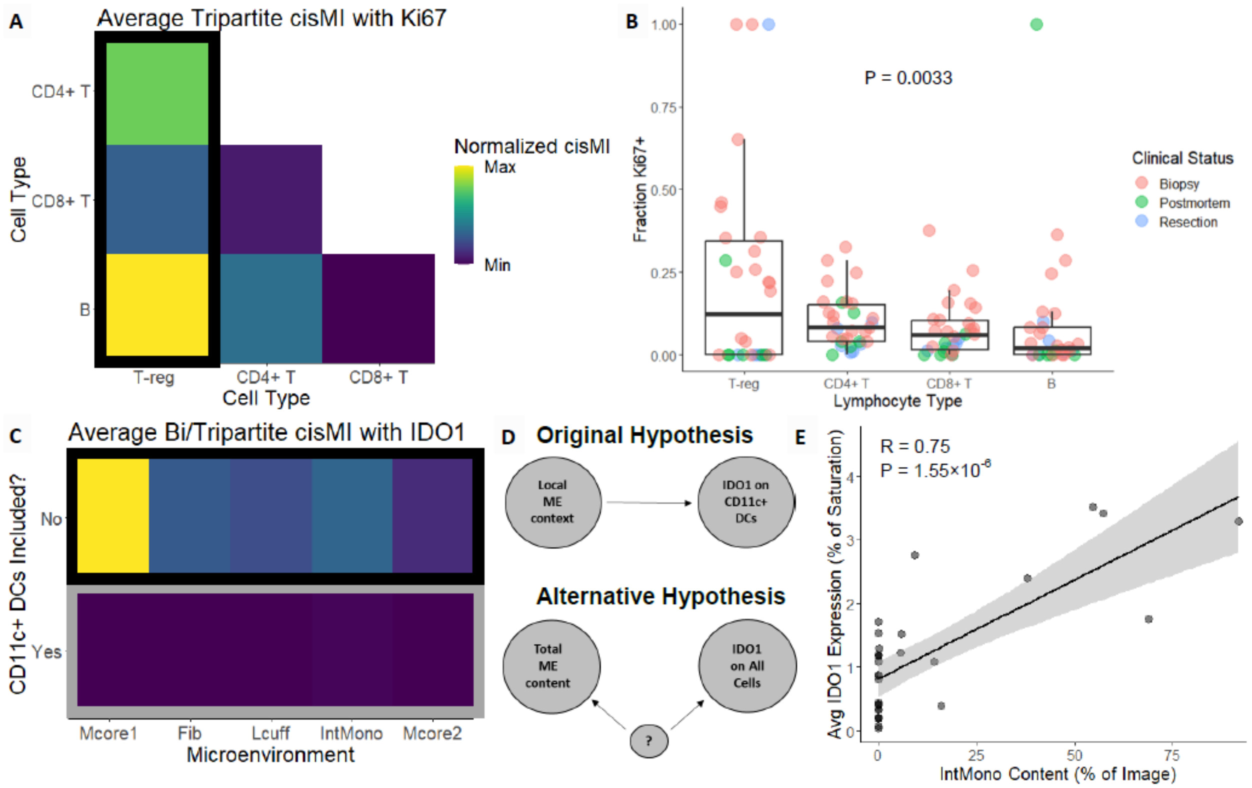

Figure S7. SPACE reanalysis of context-specific marker expression in human TB. A) Tripartite cisMI among Ki67 and pairs of lymphocyte types averaged across the 30 FoVs. B) Ki67 expression by each lymphocyte type as measured using 5µm neighborhoods in SPACE (Kruskal-Wallis test). C) Bi/tripartite cisMI among IDO1 and various MEs with or without CD11c+ DCs averaged across the 30 FoVs. D) Two hypotheses to explain higher IDO1 expression by CD11c+ DCs in some MEs than in others: direct causation of IDO1 expression changes on CD11c+ DCs by the local ME context at the cellular level, or a common driver of total ME content and total IDO1 expression irrespective of CD11c+ DCs at the FoV-level. E) FoV-wide IntMono content correlates with FoV-wide IDO1 expression, irrespective of CD11c+ DCs.
